# Novel medications for problematic alcohol use

**DOI:** 10.1172/JCI172889

**Published:** 2024-06-03

**Authors:** Markus Heilig, Katie Witkiewitz, Lara A. Ray, Lorenzo Leggio

**Affiliations:** 1 Center for Social and Affective Neuroscience, Linköping University, and Department of Psychiatry, Linköping University Hospital, Linköping, Sweden.; 2 Department of Psychology and Center on Alcohol, Substance Use and Addictions, University of New Mexico, Albuquerque, New Mexico, USA.; 3 Department of Psychology, UCLA, Los Angeles, California, USA.; 4 Clinical Psychoneuroendocrinology and Neuropsychopharmacology Section, Translational Addiction Medicine Branch, National Institute on Drug Abuse Intramural Research Program and National Institute on Alcohol Abuse and Alcoholism Division of Intramural Clinical and Biological Research, NIH, Baltimore and Bethesda, Maryland, USA.

## Abstract

Alcohol-related harm, a major cause of disease burden globally, affects people along a spectrum of use. When a harmful pattern of drinking is present in the absence of significant behavioral pathology, low-intensity brief interventions that provide information about health consequences of continued use provide large health benefits. At the other end of the spectrum, profound behavioral pathology, including continued use despite knowledge of potentially fatal consequences, warrants a medical diagnosis, and treatment is strongly indicated. Available behavioral and pharmacological treatments are supported by scientific evidence but are vastly underutilized. Discovery of additional medications, with a favorable balance of efficacy versus safety and tolerability can improve clinical uptake of treatment, allow personalized treatment, and improve outcomes. Here, we delineate the clinical conditions when pharmacotherapy should be considered in relation to the main diagnostic systems in use and discuss clinical endpoints that represent meaningful clinical benefits. We then review specific developments in three categories of targets that show promise for expanding the treatment toolkit. GPCRs remain the largest category of successful drug targets across contemporary medicine, and several GPCR targets are currently pursued for alcohol-related indications. Endocrine systems are another established category, and several promising targets have emerged for alcohol indications. Finally, immune modulators have revolutionized treatment of multiple medical conditions, and they may also hold potential to produce benefits in patients with alcohol problems.

## A spectrum of problematic alcohol use

Alcohol is one of the most commonly used psychoactive substances worldwide. Its use remains controlled in a majority of people but is also a major cause of morbidity and mortality globally, accounting for about 5% of global disease burden and close to 6% of all deaths (1, 2). Alcohol causes wide-ranging health effects (2) that can affect people even in the absence of otherwise disordered behavior. Accordingly, the WHO International Classification of Diseases, 11th edition (ICD-11) (3), defines a diagnostic category of “a harmful pattern of alcohol use,” with a lifetime prevalence in an international sample of regular alcohol users estimated at 21.6% (4).

When characteristically disordered behavior does emerge, ICD-11 defines a diagnosis of “alcohol dependence” (AD). AD is characterized as a cluster of behavioral, cognitive, and physiological phenomena that develop after repeated problematic alcohol use (3) (Table 1). In the international survey cited above, lifetime prevalence of AD was 7% (4). Excellent concordance was been found between ICD-11 AD and the corresponding diagnosis in ICD-10 as well as in the previous edition of the American Psychiatric Association (APA) Diagnostic and Statistical Manual of Mental Disorders, DSM-IV (5). The current DSM edition, DSM-5 (6), uses a broader diagnosis of “alcohol use disorder” (AUD) with mild, moderate, and severe subcategories (Table 2). Field studies have shown that moderate-to-severe AUD according to DSM-5 correlates with AD in the DSM-IV and ICD systems, while the mild category does not (4, 7). Below, we therefore use AD when referring to DSM-IV or ICD “alcohol dependence” or DSM-5 “moderate-to-severe alcohol use disorder.” Discrepancies between these diagnostic entities likely contribute to diverging views of natural history and treatment needs. At the mild end of an alcohol problem severity spectrum, rates of spontaneous remission are high, and some researchers have questioned whether a medical diagnosis and treatment are warranted (for discussion, see ref. 8). At the other end, people with AD and very heavy drinking suffer serious health consequences. In a group of European countries, those that fall in this category made up about 0.8% of people aged 15–65, accounted for about half of all liver cirrhosis cases, and had a life expectancy that was shortened by 25–31 years (9). For a discussion of remission rates, chronicity, and treatment needs in relation to the different constructs used, see ref. 8.

## Current treatment options and unmet needs

In the absence of dependence, people with harmful use respond well to brief psychological interventions from healthcare professionals, with a potential for large health benefits (1). Occasionally, pharmacotherapies may be warranted in these cases as well, e.g., as targeted administration of the opioid antagonists naltrexone or nalmefene in situations associated with risk of excessive use (10, 11). When AD has developed, screening, diagnosis, and treatment are essential for preventing or minimizing serious negative medical and social consequences. Specific behavioral as well as pharmacological treatments for AD have solid support in evidence (12), and the combination of pharmacotherapy with evidence-based behavioral interventions is superior to pharmacotherapy combined with treatment as usual (13). Despite these developments in evidence-based interventions, only about 1 in 6 people with AD ever receive treatment, and then only years after meeting diagnostic criteria (14–16). This is to a large extent the result of stigma, lack of treatment provider training, and other systemic factors such as high treatment costs, low socioeconomic status, and lack of perceived need for treatment (17). However, limitations of current treatments contribute to the treatment gap. Existing pharmacotherapies for AD provide meaningful clinical benefits, and it is important that systematic efforts are made to make sure they are provided to patients (1, 18). The hope is, however, that research will expand the treatment toolkit, allowing improved effect sizes, better opportunities for personalized treatments, and better outcomes.

Medications currently approved for the treatment of AD in Europe and the United States, as well as those with sufficient evidence to support off-label use have been reviewed (12, 18, 19) and will only be mentioned briefly here. Approved medications target three distinct mechanisms. Disulfiram, an aldehyde dehydrogenase inhibitor, results in accumulation of acetaldehyde upon alcohol intake. This alcohol metabolite is aversive at low levels and toxic at higher plasma concentrations. Naltrexone and its analog nalmefene are mu-opioid receptor–preferring (MOR-preferring) opioid antagonists thought to attenuate alcohol reward. The homo-taurine analog acamprosate exerts complex effects on glutamatergic mechanisms, but its exact mechanism of action is not known.

In addition, several medications are supported by evidence that warrants their off-label use. Topiramate, a state-dependent blocker of neuronal sodium channels, is approved for epilepsy, migraine, and obesity. It has also shown robust effect sizes in treatment of AD, although its clinical use is limited by cognitive and other side effects. Gabapentin, a ligand of α2δ voltage–dependent calcium channel subunit, is approved for treatment of focal seizures and neuropathic pain. It has also shown efficacy in several AD trials. Both these medications are recommended as second-line treatments for AD by the APA. Furthermore, varenicline, a nicotinic partial agonist approved for smoking cessation has some support for efficacy in AD. This makes it an attractive treatment option for patients with AD and nicotine use disorder. Finally, baclofen, a GABA-B agonist baclofen, is both of interest as a therapeutic in its own right and also by pointing to a therapeutic mechanism with potential yet to be exploited. It is discussed further in the section on GPCR targets.

## Outcome measures, meaningful clinical benefits, and regulatory standards

Abstinence from alcohol has long been considered the ideal outcome of AD treatment, and any drinking is associated with increased risk of morbidity and mortality (20). Abstinence from alcohol is recommended for people at the very severe end of the AD continuum, in particular those with advanced alcohol-associated liver disease, such as alcohol-induced hepatitis. This condition has a high short-term mortality, and in these patients, any drinking can lead to alcohol cirrhosis and acute liver failure. Although 15%–20% of very heavy drinkers will develop lifetime alcohol cirrhosis, the rate of alcohol-associated liver disease in the total population has been estimated at 1% or less (21).

For the majority of people who drink, and even among those who develop problems related to their alcohol use, complete abstinence may neither be required nor realistic as a treatment goal. Most people are unable to maintain abstinence following treatment (22). Furthermore, people with AD may not be interested in abstaining from alcohol use, but they may nevertheless be interested in meaningful drinking reduction goals (23). The COMBINE study, the largest trial to date evaluating candidate pharmacotherapies for AD, included 1,383 recently abstinent people with AD and assessed effects of the naltrexone or acamprosate, alone or in combination. Among the participants of this study, only 36.6% endorsed complete abstinence as their treatment goal, while 32.8% endorsed a conditional abstinence goal and 25% reported controlled drinking as their goal, and 5.6% of participants reported not having a clear goal in mind (24). Thus, it is important to offer personalized goals, including those that focus on harm reduction, for people who seek treatment (25–27).

Alternative outcomes that are commonly used in alcohol clinical trials include percentage of days abstinent, drinks per drinking day, percentage of heavy drinking days, and drinking consequences, as well as improvements in functioning and quality of life (28, 29). Drinking outcomes can be ascertained from self-reported alcohol consumption using the timeline follow-back methodology (30), considered the gold standard tool for assessing drinking outcomes in alcohol clinical trials (28). These self-reported drinking measures can be supported by biomarkers, among which phosphatidylethanol in blood (PEth) and ethylglucuronide in urine both provide measures of recent drinking (past 4 weeks and past several days, respectively) that are highly specific and both more specific and more sensitive than other commonly used lab tests, such as liver function tests (31–34). In the future, transdermal alcohol sensors may provide direct measures of alcohol consumption in near real time, but technological advances are needed before these sensors can produce reliable and valid data (35, 36).

There is substantial evidence that reductions in drinking, even short of total abstinence, are associated with clinically meaningful improvements in health and functioning (37–42). There is also a growing consensus in the field that reduced drinking is an acceptable treatment outcome for AD (23, 43). The WHO defines four sex-specific risk levels of drinking that are useful for defining treatment targets in clinical care (Table 3) (44). Recent evidence from population-based and clinical samples indicates that drinking reductions of at least one- or two-WHO risk drinking levels are associated with clinically and statistically significant improvements in mental health (40, 41, 45), physical health (38, 39, 42), reduced risk of liver disease and improvements in liver function (39, 42), improvements in regional gray matter volume (46), and significant medication effect sizes as compared with placebo equivalents (47, 48).

**Figure 1:**
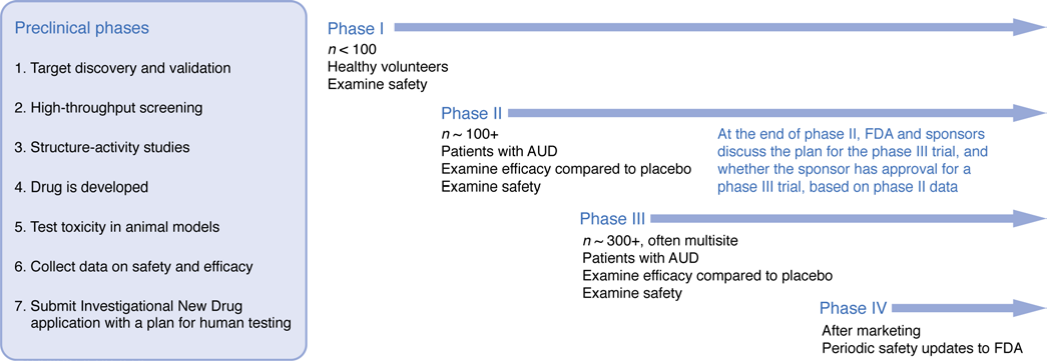
Schematic of approval process for medication development, as outlined by the US FDA.

Despite the clear value of drinking reductions in improving health and functioning, and preference among most people with AD who desire drinking reduction goals, the US FDA and the European Medicines Agency (EMA) continue to promote abstinence as the ideal treatment target (49, 50). However, in recognition of the fact that many people do not achieve abstinence, and that drinking reductions may be more valued by patients with AD, the FDA has updated their guidance for alcohol medication development to allow no heavy drinking days as a binary endpoint in alcohol clinical trials (49). In this context, heavy drinking days are defined as more than 3 drinks per occasion for women and more than 4 drinks per occasion for men. Similarly, the EMA now also allows for intermediate harm reduction goals as primary endpoints for alcohol clinical trials, with reductions in total consumption of alcohol per month and reductions in the number of heavy drinking days (defined as more than 60 grams of pure alcohol in men and 40 grams in women) approved as primary outcomes (50). The EMA also allows secondary responder endpoints of the proportion of people with a 50%, 70%, and 90% reduction in alcohol consumption, the proportion of patients achieving sustained abstinence, and the proportion of patients who achieve at least a two-level reduction in WHO risk drinking levels. The FDA is currently considering a proposal to adopt reductions in WHO risk drinking levels as an endpoint for alcohol clinical trials in the United States (51). See Figure 1 for an outline of the medications approval process by the FDA.

## GPCR targets

### GABA-B receptor–positive allosteric modulators.

γ-Amino butyric acid (GABA), the main inhibitory neurotransmitter of the mature mammalian brain, signals through ligand-gated chloride channels (GABA-A) and G_i_/_o_-coupled metabotropic receptors (GABA-B). The latter, assembled as dimers of GABA-B1 and -B2 subunits, hyperpolarized cells, expressing them by activating G protein–coupled inwardly rectifying potassium channels and inhibited adenylyl-cyclase activity. These actions result in a decrease of excitability or inhibition of neurotransmitter release. The diverse actions of pre- and postsynaptic GABA-B receptors have been reviewed (52). Baclofen remains the prototypical orthosteric GABA-B agonist. It was developed in the early 1960s for treatment of epilepsy and failed to show efficacy on this indication, but was it approved in 1977 for spasticity.

Foundational work by Colombo, Addolorato, and colleagues in the early 2000s provided initial support for the ability of baclofen to suppress alcohol withdrawal and intake in rats and to reduce alcohol drinking and craving in patients with AD (53–55). This was followed by a seminal clinical trial in which baclofen robustly promoted abstinence over 12 weeks in patients with severe AD and liver cirrhosis (56). Multiple clinical trials then evaluated the efficacy of baclofen in AD. Results were variable, but a meta-analysis found overall support for the efficacy of baclofen and identified dependence severity as an important cause of heterogeneity in results (19). Additional support for overall efficacy of baclofen has since been obtained; it was also found that optimal dosing may differ between men and women, with women showing higher sensitivity to dose-limiting sedative side effects (57, 58).

Baclofen is an orthosteric agonist, and its chronic use predictably results in tolerance. In many cases, this necessitates dose escalation, in turn increasing the risk of serious adverse events. With appropriate monitoring and precautions, off-label use of baclofen can be justified in those with AD, especially in patients with high dependence severity, and in those with liver disease. However, safety concerns limit the use of baclofen and its approval for AD (59). Positive allosteric modulators (PAMs) potentially offer a strategy for amplifying GABA-B receptor signaling, while minimizing these undesirable effects. PAMs bind to a distinct site on the receptor, and their binding does not directly activate downstream signaling. Instead, binding at an allosteric site shifts the receptor protein conformation toward a state in which the affinity and therefore the response to subsequent agonist binding is amplified (60). In animal models, GABA-B PAMs are devoid of tolerance (61), offering a path forward for GABA-B activation as a therapeutic mechanism in AD (62). Among GABA-B PAMs discovered, ADX71441 (63) and ASP8062 (64) showed promising preclinical activity and were nominated as clinical candidates. For ADX71441, a toxicity signal was identified and led to termination of its development, but back-up molecules are currently in development. ASP8062 cleared toxicology, and it has completed evaluation in a multisite human laboratory study sponsored by the US National Institute on Alcohol Abuse and Alcoholism (NCT05096117). This study, carried out in 60 participants with moderate-to-severe AUD according to DSM-5, assessed craving responses as a biomarker. It was completed in 2023, but results are not yet available.

Recent animal studies have shed light on mechanisms that may contribute to the efficacy of GABA-B activation in AD. Dysregulation of GABA transmission in the central nucleus of amygdala appears to promote two behaviors that are at the core of addictive disorders: choice of alcohol over natural rewards and continued use despite negative consequences. Both these behaviors were rescued by GABA-B receptor activation (65–67). If safe and well-tolerated GABA-B PAMs can be brought to the clinic, these findings should inform their use. They emphasize that, in addictive disorders, medications should be viewed as tools to facilitate a shift of behavior away from substance use and toward healthy options, rather than simply to suppress substance use (68, 69).

### κ-Opioid receptor antagonists.

Endogenous opioid systems play diverse roles in addictive disorders (70, 71). MORs, preferentially activated by β-endorphin (BEND), contribute to the “liking” of multiple rewards, including alcohol. In contrast, κ-opioid receptor (KOR) activation by dynorphin mediates stress reactivity and negative emotionality (72). In animal studies, prolonged brain alcohol exposure results in increased KOR activity that underlies both aversive properties of alcohol withdrawal and attenuated mesolimbic dopamine signaling (73, 74). Together, this results in a combination of negative affect and reward deficit that promotes alcohol use through negative reinforcement. In preclinical models, KOR antagonism blocks stress-induced relapse (75, 76), while administration of a KOR agonist triggers it. KOR activation promotes alcohol taking and relapse to alcohol seeking through actions in central nucleus of amygdala and the bed nucleus of stria terminalis (77–81).

These and other preclinical findings provided compelling validation of KOR antagonism as a mechanism for treatment of AD, but translation was long prevented by a lack of KOR antagonists with drug-like properties. The prototypical tool compound, nor-binaltorphimine (nor-BNI), has effects that outlast its dissociation from the receptor, due to activation of c-Jun N-terminal kinase (82). This was also found with a clinical candidate, JDTic (82), that additionally turned out to be cardiotoxic (83). More recently, the discovery of safe, short-acting KOR antagonists has allowed the therapeutic potential of KOR antagonists to be examined. A key advance was the discovery of aticaprant (successively designated LY-2456302, CERC-501, and JNJ-67953964) (84). In phase I studies, aticaprant was safe and well tolerated in volunteers without cocaine dependence and among individuals with cocaine dependence (85–87).

Despite an overwhelming body of preclinical evidence identifying KOR signaling as a mechanism behind stress-induced substance use and relapse, an initial laboratory smoking study evaluated aticaprant for its effects on smoking-related behaviors in the absence of stress and was thoroughly negative (88). A trial better aligned with insights from preclinical research was subsequently carried out under the Fast-Fail initiative of the US National Institute of Mental Health (89). Taking note of observations that KOR signaling produces a “reward-deficit,” this study recruited patients with anxiety or depression who also showed anhedonia, i.e., a decreased ability to experience pleasure. In agreement with the hypothesis tested, aticaprant showed beneficial effects on a fMRI biomarker of brain reward responses and reduced self-reported anhedonia (90). A phase II trial of aticaprant for depression was then carried out by Janssen Pharmaceuticals and was positive (91). Similar results were obtained with navacaprant, a KOR antagonist originally developed by BlackThorn Therapeutics and subsequently acquired by Neumora (NCT04221230). Phase III programs to develop KOR antagonists for anhedonic depression are currently pursued by these and other companies.

Based on the role of KORs and negative affect in AD, Domi and colleagues carried out studies to assess the potential of aticaprant as a clinical candidate in AD using rat models of alcohol-related behaviors (92). Similar to what is observed in humans, a marked increase in anxiety-like behavior is seen in rats during alcohol withdrawal, and systemic administration of aticaprant resulted in a complete reversal of this behavior. Another clinical characteristic of AD is escalation of the alcohol amounts taken. In rats, aticaprant did not influence baseline alcohol self-administration under nonescalated conditions but reversed the escalation seen following prolonged intermittent access to alcohol. Finally, in the reinstatement model of relapse to alcohol seeking, aticaprant selectively decreased reinstatement induced by a stressor. In contrast, reinstatement triggered by alcohol-associated stimuli was unaffected.

Overall, the activity profile observed in these experiments was consistent with that predicted by pioneering research on the role of KOR activation in alcohol-related behaviors carried out by others (72, 73, 78, 80, 81, 93). The findings indicate that aticaprant, and presumably other KOR antagonists, has a potential to produce beneficial clinical effects in AD by acting on negative affective states that promote alcohol use and relapse. This mechanism is attractive in its own right but can additionally be expected to complement that of naltrexone, an approved AD medication thought to inhibit alcohol reward and relapse induced by alcohol-associated stimuli (94). Because these two mechanisms target different components of AD, combining them has the potential to be additive and result in improved effect sizes. The fact that KOR antagonism reduces anhedonia and negative affective states also suggests that it is likely to meet with good patient acceptance. This would be an important feature of KOR antagonism as a treatment per se, but it could potentially also improve patient compliance with a combined treatment.

## Emerging neuroendocrine-based mechanisms and therapeutic targets

**Figure 2:**
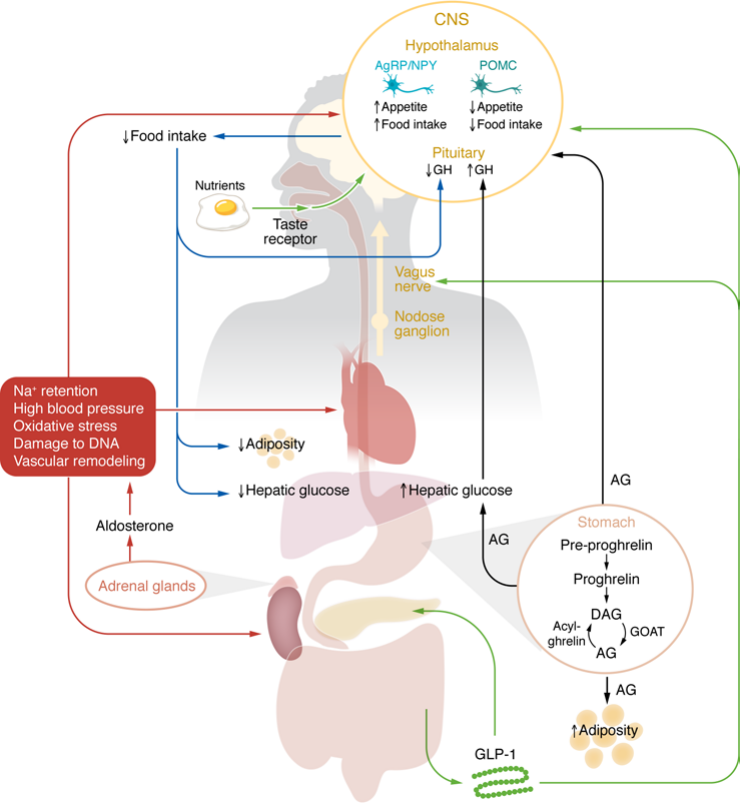
Schematic of putative entero- and neuroendocrine mechanisms that can be targeted by medications for AD. AG, acyl-ghrelin (also known as ghrelin); AgRP, agouti-related peptide; CNS, central nervous system; DAG, des-acyl-ghrelin; DNA, deoxyribonucleic acid; GH, growth hormone; GLP-1, glucagon-like peptide-1; GOAT, ghrelin O-acyltransferase; NPY, neuropeptide Y; POMC, pro-opiomelanocortin.

Bidirectional brain-body interactions are involved both in normal psychological function and in psychiatric disorders and may be particularly important in addiction (95, 96) (Figure 2). Accordingly, there is growing evidence that alcohol-related behaviors can be modulated, directly or indirectly, via neuroendocrine mechanisms. Growing research has investigated the role of peripheral neuroendocrine signaling in AD and holds a potential for identifying new medication targets. The gut-brain axis is a prominent example of these advances. Physiologically, hormones secreted by enteroendocrine and adipose cells are released in response to nutrient availability and communicate metabolic and nutrient state to the brain. This, in turn, directs appetite, food seeking, food intake, and food choice, based on energy needs (97, 98). Beyond energy homeostasis, these neuroendocrine systems are increasingly recognized to play a role in mechanisms of stress, emotionality, and rewarding properties of food and addictive drugs, including alcohol (99). Ghrelin and glucagon-like peptide-1 (GLP-1) are two examples of gut-brain neuroendocrine systems that have been implicated in alcohol seeking and dependence.

### Ghrelin and its receptor.

Ghrelin, a peptide hormone, is primarily produced by enteroendocrine cells of the stomach and promotes appetite through hypothalamic mechanisms (100). A seminal study in mice reported that ghrelin increased whereas pharmacological blockade or genetic knockout of the ghrelin receptor (growth hormone secretagogue receptor [GHSR]) reduced alcohol drinking and reward (101). Multiple studies subsequently provided support for these findings (reviewed in ref. 102). Human studies showed a positive relationship between ghrelin levels and alcohol craving, drinking, relapse, subjective responses to alcohol, and brain activity in response to alcohol cues (reviewed in ref. 102). These correlational studies did not establish causality, but subsequent experimental findings supported a causal role of ghrelin to promote alcohol seeking. Specifically, two placebo-controlled randomized experimental medicine studies in people with AD found that intravenous ghrelin administration increased cue-induced alcohol craving in a bar-like setting, increased alcohol self-administration in a progressive-ratio procedure, and modulated brain activity in response to alcohol reward anticipation during an fMRI neuroimaging procedure (103, 104). These findings provided a validation for GHSR blockade as a target for pharmacotherapy in AD, but progress was limited by a lack of phase II–ready GHSR blockers available for clinical development in people with AD. Recently, the GHSR inverse agonist PF-5190457 was found to be safe and well tolerated in healthy volunteers (105). A phase Ib study then supported safety and tolerability of PF-5190457 when coadministered with alcohol in people who are heavy drinkers and provided suggestive evidence for reduction of cue-induced alcohol craving and attention to alcohol cues in a bar-like setting (106).

### GLP-1.

GLP-1, a peptide hormone generated by posttranslational processing of the glucagon precursor, is secreted by enteroendocrine L-cells in the intestine upon food consumption. In contrast to ghrelin, it has been postulated that boosting rather than blocking GLP-1 signaling may lead to reductions in alcohol use (107). An association, replicated in independent cohorts, has been found between rs6923761, a Gly1168Ser SNP at the locus encoding the GLP-1 receptor (GLP-1R), and several alcohol-related phenotypes (108). Furthermore, administration of GLP-1 itself or GLP-1R agonists (GLP-1RAs), such as exenatide and liraglutide, reduced alcohol drinking and other alcohol-related behaviors in rodents (109) and nonhuman primate models (110).

The availability of GLP-1RAs approved for clinical use in diabetes and obesity potentially provides a path to repurposing them for AD. To date, however, data unequivocally supporting clinical efficacy of GLP-1As on this indication are lacking. A double-blind, placebo-controlled AD trial carried out with exenatide did not provide support for efficacy on the primary alcohol drinking outcomes. However, an effect was observed on fMRI-based cue craving in the ventral striatum and septal area. The study also presented a secondary analysis that suggested that exenatide significantly reduced heavy drinking days and total alcohol intake in participants with BMIs of more than 30 kg/m^2^, while producing an increase in participants with BMIs of less than 25 kg/m^2^ (111).

The newer GLP-1RA semaglutide has shown greater efficacy than exenatide in both diabetes and obesity, the two conditions for which it is currently approved. This has increased interest in testing semaglutide in AD. Recent mouse and rat experiments have shown robust effects of semaglutide on alcohol drinking outcomes (112, 113). Many anecdotal reports of people taking semaglutide for diabetes or obesity are in line with these findings and have received considerable media attention. Clearly, however, well-designed randomized controlled trials (RCTs) are needed before the efficacy of semaglutide for AD can be determined (114).

### Mineralocorticoid signaling.

The aldosterone/mineralocorticoid receptor (MR) system mediates bidirectional neurocardiovascular communication. Observational studies have shown a correlation between circulating aldosterone levels and alcohol craving and drinking in patients with AD (115, 116). In addition, a negative correlation between MR expression in the amygdala, a key region in AD mechanisms, and alcohol drinking was found in rat and nonhuman primate models of AD (116). Although correlational, these findings prompted the question of whether MR may represent a novel target for AD treatment.

Consistent with this hypothesis, the nonselective MR antagonist spironolactone was recently shown to reduce alcohol drinking in mouse and rat models (117, 118). Spironolactone has been used clinically for decades to treat hypertension, edema, and chronic heart failure. This provided an opportunity to retrospectively analyze electronic medical records for its potential effects on alcohol use. Two pharmacoepidemiological studies using propensity score matching independently addressed this question. These studies analyzed data from two electronic medical records systems that captured diverse clinical populations, patients in primary care and patients in the Veterans Administration medical system, respectively. The findings provided converging evidence that spironolactone prescription for any indication was associated with a reduction in alcohol use. This association was dose dependent and was stronger in people with higher severity of AD (118, 119). These pharmacoepidemiological findings provide a strong rationale for RCTs to directly evaluate the potential efficacy of spironolactone in AD.

## Immune modulators

Accumulating data suggest that immune mechanisms are critically involved in the development and maintenance of AD (120, 121). Alcohol promotes systemic production of proinflammatory cytokines that ultimately impact brain function. For instance, systemic inflammation is induced by alcohol when it acts on peripheral immune receptors in the gut (122), allowing inflammation triggering molecules to leak into the bloodstream in a process often termed “leaky gut” (123). In addition, direct actions of alcohol on the brain cause local release of proinflammatory molecules (124, 125). It should be noted that neuroinflammation can be adaptive and promote repair following neuronal injury but that it is frequently maladaptive, for instance, when mounted in response to chronic social stressors (126). When neuroinflammatory responses are excessive or prolonged, they can contribute to psychiatric and physical disorders (127), and this seems to be the case with sustained heavy alcohol use. While the interplay between immunity and AD is reviewed in detail elsewhere (128), we will next describe advances in pharmacotherapy for AD that target the immune system (129).

### TLRs.

TLRs are members of the IL-1 receptor/TLR superfamily. They share with other proinflammatory cytokine receptors intracellular signaling pathways that converge on NF-κB, an inducible transcription factor that regulates the expression of proinflammatory cytokines involved in innate immune responses. Alcohol and stress are thought to modify TLR signaling in corticolimbic circuits, in turn promoting the progression of AD (130). Opioid antagonists, including naltrexone and naloxone, can block TLR4s (131), and nalmefene was found to inhibit TLR4 signaling, decrease alcohol-induced inflammation, and reduce associated binge drinking in mice (132). Nevertheless, it remains unclear to what extent TLR4 antagonism contributes to the effects of opioid antagonists on drinking, given the well-established primary mechanism of MOR blockade on alcohol intake.

Given the key role of NF-κB signaling as a link between neuroimmune mechanisms and AD, therapies bypassing TLR binding and acting directly on NF-κB may also hold promise as AD therapeutics. This is supported by preclinical findings with immunotherapies such as sulfasalazine and TPCA-1 that act on NF-κB through IKKβ, an inhibitor of the NF-κB kinase subunit β. These inhibitors of IKKβ have been shown to decrease alcohol consumption and preference in mice (133). For instance, amlexanox, an NF-κB inhibitor (134), reduced alcohol preference in mice (135). While several TLRs compounds have demonstrated safety for other medical conditions, they have yet to be tested for AD.

### Phosphodiesterase inhibitors.

Phosphodiesterases (PDEs) regulate intracellular levels of cAMP and cGMP. PDEs modulate the cAMP PKA pathway, which regulates responses to acute and chronic alcohol exposure (136). PDE inhibitors have been studied using animal models of AD, with particular focus on PDE4 inhibition (137). Rolipram, a PDE inhibitor that reached clinical trials for neurodegenerative disorders and depression (138), reduced alcohol intake and preference in mice (139) and rats (140) but was not well tolerated in humans, where it caused vomiting and headaches. Other PDE4 inhibitors have been evaluated in preclinical models, including mesopram, piclamilast, and CDP840 (141). Roflumilast was found to decrease alcohol intake and preference in mice (142).

Apremilast, a partial competitive PDE4 inhibitor, is FDA and EMA approved for the treatment of psoriasis and has acceptable safety and tolerability (143). In animals, apremilast reduced alcohol intake and preference but did not modify sucrose preference, indicating that its effects may be alcohol specific (141). In a recent study, apremilast reduced alcohol intake in nontreatment seeking individuals with AD over the course of 11 days of treatment (144). A promising pattern of preclinical and early-stage human findings has also been found with another the PDE inhibitor. Ibudilast, a preferential inhibitor of PDE3A, PDE4, PDE10A, and PDE11A, was found to reduce drinking and relapse in multiple animal models of alcohol related behaviors (145). In nontreatment seekers with AD, ibudilast decreased tonic craving for alcohol and improved mood following alcohol cue and stress exposure (146). In a separate clinical study, ibudilast reduced rates of heavy drinking and neural alcohol cue–reactivity over a 2-week treatment period (147). A secondary analysis suggested that individuals with elevated C-reactive protein levels at baseline had the best clinical response to ibudilast (148). In summary, both apremilast and ibudilast have preclinical and early human efficacy studies with positive results for treating AD. Clinical trials have recently been completed or are underway to determine their therapeutic potential.

### PPARs.

PPARs are thought to modulate pathways involved in NF-κB activation and nitric oxide production and inhibit expression of TNF-α (148). PPAR agonists have antiinflammatory activity, are approved as treatments for insulin resistance in diabetes and hyperlipidemia (149), and show promise as immune therapies for AD and other central nervous system diseases (150). Preclinical work has implicated PPARs in regulation of alcohol intake, stress-induced alcohol seeking, and withdrawal (150). The PPARγ agonist pioglitazone reduced voluntary drinking, lever pressing for alcohol, and reinstatement of alcohol-seeking behavior, but did not prevent cue-induced relapse in animal models (151). Pioglitazone combined with naltrexone resulted in further attenuation of alcohol intake in alcohol preferring rats (152). Based on these findings, an experimental medicine study was initiated in people with AD but was terminated because several patients randomized to pioglitazone developed myopathy (153). Among those who completed the study, cue-induced alcohol craving was increased, rather than decreased, by pioglitazone. Other studies with pioglitazone in AD are currently ongoing (NCT05107765; NCT03864146).

Fenofibrate (targeting PPARα), tesaglitazar (dual agonist: PPARα/γ), and bezafibrate (pan agonist: PPARα/γ/δ) have also been tested in animals (154, 155). While fenofibrate and tesaglitazar produced long-lasting reductions in alcohol intake, bezafibrate produced null findings, and the effects of PPAR agonists were dependent on drinking paradigm, sex, and genotype (139, 156).

PPAR agonists may influence alcohol use through central as well as peripheral modulation of immune function (122). PPARα agonists with actions in the periphery have been tested in animals. Oleoylethanolamide, an endocannabinoid-like endogenous compound with antiinflammatory properties mediated by PPARα activation has been shown to prevent depressive-like behavior induced by binge administration of alcohol in rats (157). The clinical approval of fenofibrate for treatment of hyperlipidemia offers a potential path for developing PPARα agonism for AD by repurposing this medication.

### Microglia modulation.

Microglia have been shown to regulate escalation of drinking and AD-induced changes in neuronal function (158). Minocycline, a broad-spectrum antibiotic that crosses the blood-brain barrier, is a microglial attenuator that alters immune responses and cytokine expression in the brain and periphery (159). Preclinical studies have found that minocycline modestly reduced alcohol intake in a free-choice voluntary drinking model (160), reduced alcohol intake in adult but not adolescent mice (161), and reduced alcohol-induced sedation, withdrawal-related anxiety, and alcohol reinstatement (162). However, a recent study that tested minocycline for alcohol-related effects in people found no beneficial effect of a short-term minocycline treatment on inflammation or subjective response to alcohol (163).

### Other immune pharmacotherapies.

Endogenous neuroactive steroids, termed “neurosteroids,” are synthesized in the brain and modulate several pathways with the potential to target AD symptomatology, including GABA_A_R, TLR, and corticotropin-releasing factor signaling (164). Dutasteride, a 5α-reductase inhibitor, is FDA approved for treatment of benign prostate hyperplasia due to its ability to prevent conversion of testosterone to the more potent dihydrotestosterone but also inhibits neurosteroidogenesis. A human laboratory study found that dutasteride reduced the sedative effects of alcohol and heavy drinking days (165). Cannabidiol (CBD), a nonpsychoactive component of the cannabis plant, is a plausible therapeutic for AD (166). CBD exhibits neuroprotective effects by interacting with the body’s endocannabinoid system. CBD attenuated alcohol-induced increases in liver enzymes, mRNA expression of the cytokines TNF-α and IL-1β, and several chemokines (167), suggesting that CBD’s ability to prevent liver damage is partially attributable to immune processes. Currently, multiple trials of CBD for AD are underway based on the premise of its antiinflammatory properties and potential for rapid uptake in clinical settings (168).

## Concluding remarks

Large unmet treatment needs remain in the area of AD. In the short term, the greatest potential for making a dent in alcohol-related harm is by addressing the issues causing low clinical utilization of medications that are already approved or that can be used off-label based on available evidence. In the medium and long term, however, these efforts will be facilitated by expanding the treatment toolkit available to patients and providers. We have reviewed numerous, diverse biological mechanisms with potential to target toward this goal. Due to space limitations, our Review is by no means exhaustive, and additional mechanisms that may show promise exist. Among these, psychedelic treatments attract extensive public attention, and positive RCT results have been reported both with the noncompetitive NMDA antagonist ketamine (169) and the classical psychedelic 5HT2A agonist psilocybin (170). Work in this domain has recently been reviewed (171). However, interpretation is complicated by methodological challenges related to expectancy effects and consistent functional unblinding, as also discussed in the context of depression (172). Another potentially promising mechanism in clinical development is modulation of glutamatergic transmission (173), where a novel mGluR5 negative allosteric modulator is currently evaluated in a human lab study (NCT04831684).

Ongoing exploration of these diverse biological mechanisms holds the promise of improved opportunities for personalized treatments with better patient acceptance. This diversity also poses major challenges when it comes to prioritizing targets and medication candidates for resource demanding clinical trials. One of the greatest scientific challenges ahead is to develop effective models for progression from preclinical target validation, through human laboratory-based efficacy biomarkers, to clinical trials that can establish efficacy (174).

## Figures and Tables

**Table 3:**
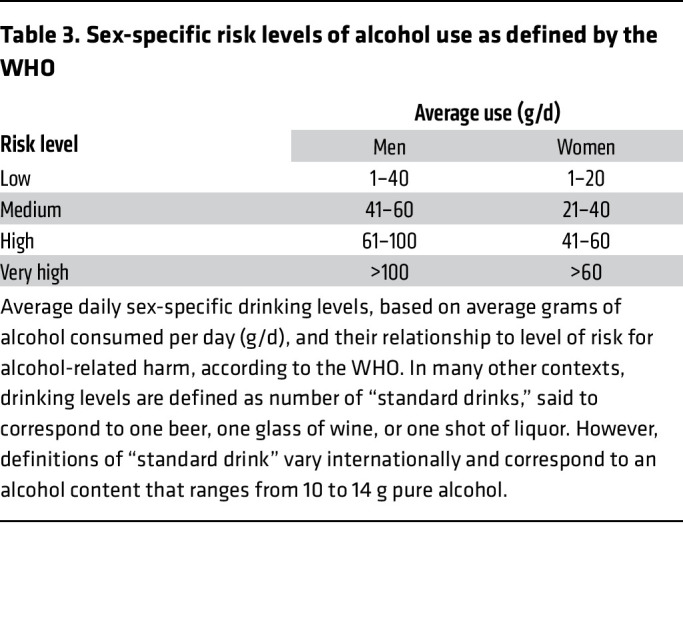
Sex-specific risk levels of alcohol use as defined by the WHO

**Table 2:**
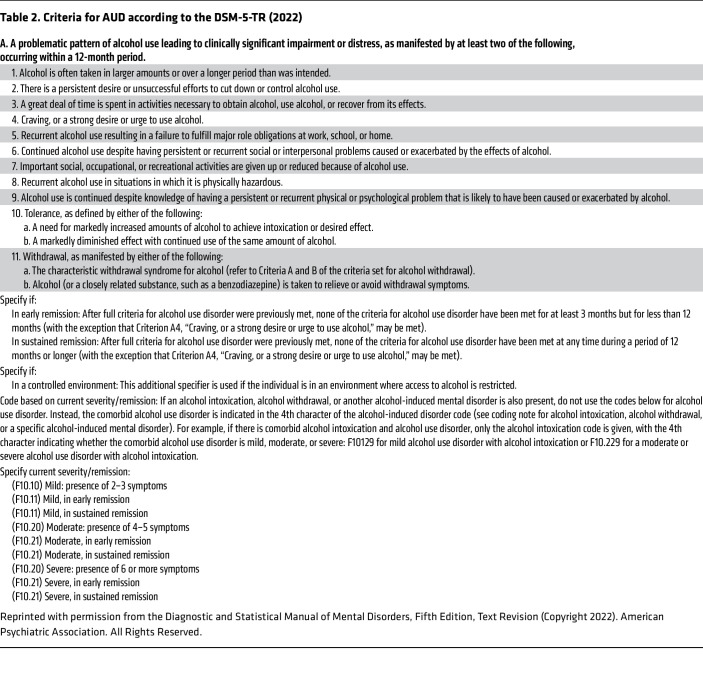
Criteria for AUD according to the DSM-5-TR (2022)

**Table 1:**
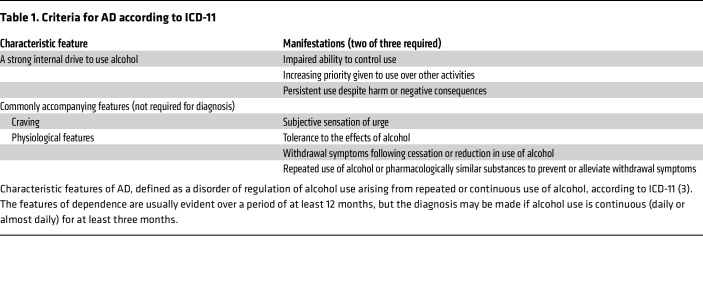
Criteria for AD according to ICD-11

## Version 1 06/03/2024

Electronic publication
